# Levetiracetam Ameliorates L-DOPA-Induced Dyskinesia in Hemiparkinsonian Rats Inducing Critical Molecular Changes in the Striatum

**DOI:** 10.1155/2015/253878

**Published:** 2015-01-27

**Authors:** Huan Du, Shuke Nie, Guiqin Chen, Kai Ma, Yan Xu, Zhentao Zhang, Stella M. Papa, Xuebing Cao

**Affiliations:** ^1^Department of Neurology, Union Hospital, Tongji Medical College, Huazhong University of Science and Technology, 1277 Jiefang Avenue, Wuhan 430022, China; ^2^Department of Neurology, Sanmenxia Central Hospital, Sanmenxia 472000, China; ^3^Department of Neurology, Renmin Hospital, Wuhan University, Wuhan 430060, China; ^4^Yerkes National Primate Research Center, Department of Neurology, Emory University School of Medicine, Atlanta, GA 30329, USA

## Abstract

L-DOPA-induced dyskinesias (LID) remain a major problem of long-term therapy of Parkinson's disease. Levetiracetam, a new antiepileptic drug, has been shown to reduce LID, but the mechanisms underlying its effects are unknown. In this study, we assessed the effect of levetiracetam on key mediators of LID in rats with 6-hydroxydopamine (6-OHDA) lesions. Following chronic administration of L-DOPA (12 mg/kg, twice daily for 14 days), rats developed abnormal involuntary movements (AIMs), but co-administration of levetiracetam (15, 30, and 60 mg/kg) with equivalent L-DOPA dosing significantly reduced AIMs scores in a dose dependent manner. The effects of levetiracetam were associated with changes in striatal expression of ΔFosB, phosphorylated extracellular signal-regulated kinases 1 and 2 (p-ERK1/2), and phosphorylated cAMP-regulated phosphoprotein of 32 kDa (p-DARPP-32). These data support that levetiracetam acts at multiple sites in the pathogenetic cascade of LID, and that further understanding of these actions of antiepileptics may contribute to developing new LID therapies.

## 1. Introduction

Dopamine replacement therapy with L-3,4-dihydroxyphenylalanine (L-DOPA), still the most effective treatment for Parkinson's disease (PD), is commonly associated with disabling long-term motor complications, including response fluctuations and L-DOPA-induced dyskinesias (LID) [1]. LID are involuntary choreic and dystonic movements that occurred as early as following 3–6 years of L-DOPA treatment in 40% of PD patients [2]. Furthermore, LID develops in the large majority of patients over the course of chronic exposure to L-DOPA. Available medical treatments for LID produce slight benefits, and in many cases the management of LID ultimately relies on DBS surgery.

Although the pathogenesis of LID remains unclear, it is believed that both disease progression and nonphysiologic pulsatile dopamine stimulus contribute to the associated changes in various signaling pathways and basal ganglia circuits [3, 4]. Physiologic and molecular studies have shown hypersensitivity of D_1_ receptor-mediated responses in the direct striatal output pathway leading to imbalance of striatal outputs [5]. Moreover, studies of advanced Parkinsonian monkeys have shown abnormal reversal of dopamine responses in direct and indirect output neurons indicating imbalanced discharges across neurons within each pathway [6]. In the direct pathway, increased activation of cAMP-dependent signaling and PKA through D_1_ receptors leads to increased phosphorylation of various downstream proteins, such as ERK1/2 (extracellular signal-regulated kinases 1 and 2) and DARPP-32 (dopamine- and cAMP-regulated phosphoprotein of 32 kDa) [7, 8]. In addition, transcription factors expressed with chronic events such as ΔFosB (a truncated splice variant of FosB) are overexpressed in the striatum of rodents and primates with dyskinesias [9, 10]. Pharmacological or genetic interventions to reduce the levels of these proteins can attenuate LID, as shown in DARPP-32-deficient mice or after inhibition of PKA in rats [11, 12]. Furthermore, the transgenic overexpression of ΔFosB reproduces AIMs in hemiparkinsonian rats without chronic exposure to L-DOPA [13].

Levetiracetam ((S)-a-ethyl-2-oxo-pyrrolidine acetamide, LEV) is an antiepileptic drug that reduces LID in the macaque MPTP model of PD [14]. The action mechanism of LEV seems to be nonconventional for its class because unlike other antiepileptic drugs LEV may predominantly bind to the synaptic vesicular protein SV2A. Additionally, coadministration of L-DOPA and LEV following one-week drug holiday in primates demonstrated significant reduction of LID pointing to effects on L-DOPA priming for LID [15]. However, levetiracetam action on LID remains elusive. Here, we tested levetiracetam effects on those molecules known to be involved in LID mechanisms in rats with unilateral 6-OHDA lesions of the striatonigral pathway. Levetiracetam had significant regulatory effects on ΔFosB, p-ERK1/2, and p-DARPP-32 expression, which correlated with reduction of abnormal involuntary movements (AIMs) demonstrating the specific effects of this antiepileptic drug on dyskinesia mechanisms.

## 2. Materials and Methods

### 2.1. Unilateral 6-OHDA-Lesioned Model

Male Sprague-Dawley (SD) rats were bought from the Experimental Animal Center of Tongji Medical College at Huazhong University of Science and Technology (200–250 g), housed in controlled environment (12 hrs light/dark cycle, 22 ± 2°C, 55 ± 5% relative humidity) and allowed free access to food and water. All of the experimental protocols were approved by the Institutional Animal Care and Use Committee of Huazhong University of Science and Technology (HUST). Rats were anesthetized with 10% chloral hydrate (0.5 mL/100 g, i.p.) and injected unilaterally in the medial forebrain bundle with 4 *μ*L of 6-OHDA (2 mg/mL, dissolved in saline/ascorbic acid, Sigma-Aldrich, St. Louis, MO) following stereotaxic coordinates (AP + 4.0, L + 1.3, DV-8.4 mm from the interaural line) at a rate of 0.5 *μ*L/min using a micropump [16].

**Figure 1:**
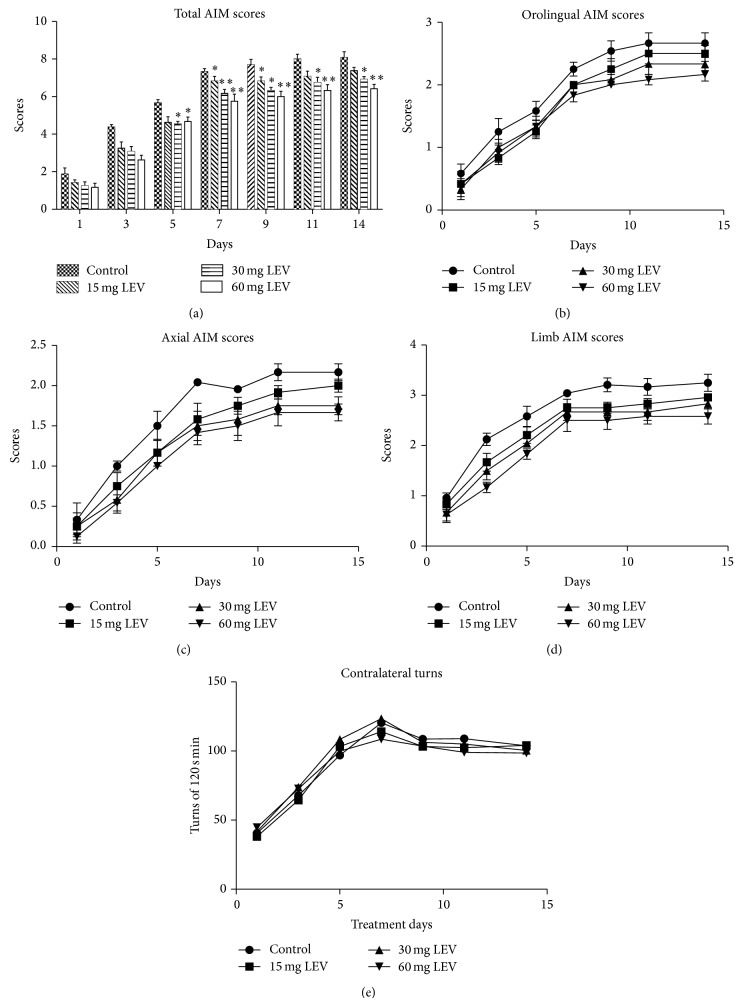
Effects of levetiracetam on abnormal involuntary movements (AIMs) induced by L-DOPA in hemiparkinsonian rats. Animals were assessed on days 1, 3, 5, 7, 9, 11, and 14 following L-DOPA methyl ester (12 mg/kg) plus benserazide-HCl (3 mg/kg) (i.p.). Two hours prior to each L-DOPA injection, animals received levetiracetam 0 (control group, vehicle injection), 15, 30, or 60 mg/kg by gavage. Total AIMs scores (a) include orofacial (b), axial (c), and limb (d) AIMs scores. Rotational behavior (contralateral turns, (e)) shows that levetiracetam induced no changes in the antiparkinsonian action of L-Dopa. Values are mean ± SEM. ^*^ *P* < 0.05 and ^**^ *P* < 0.01 versus control group (*n* = 10 per group; LEV: levetiracetam).

Three weeks after surgery, rats were injected subcutaneously with a subthreshold dose of apomorphine (0.05 mg/kg) to test for a rotational response indicative of supersensitivity developed with full denervation. Rats exhibiting an average of ⩾7 turns/min were selected for the study and randomly divided in four groups of 10 rats each: (A) control group, (B) 15 mg levetiracetam group, (C) 30 mg levetiracetam group, and (D) 60 mg levetiracetam group (see Figure 1).

### 2.2. Drug Treatment

Rats were pretreated by gavage with levetiracetam (15, 30, or 60 mg/kg) as previously described [14] and two hours later injected intraperitoneally with L-DOPA methyl ester (12 mg/kg) plus benserazide-HCl (3 mg/kg) dissolved in saline (Sigma-Aldrich, Madrid, Spain) twice daily (9 AM and 5 PM) for 14 days [17]. The control group received saline before L-DOPA treatment.

### 2.3. Behavioral Measurements

Animals were assessed on days 1, 3, 5, 7, 9, 11, and 14 following a challenge of L-DOPA methyl ester (12 mg/kg) plus benserazide-HCl (3 mg/kg) (i.p.). Rats were assessed individually at 30, 60, 90, and 120 min after the L-DOPA injection using an adaptation of the AIMs Scale [18]. The four subscales (oral dyskinesia, limb dyskinesia, axial dystonia, and contraversive rotation) were used to score: (1) orolingual dyskinesia, stereotyped jaw movements, and tongue protrusion, (2) forelimb dyskinesia, repetitive rhythmic jerks, and contralateral to the lesion side, (3) axial dystonia, contralateral twisted posturing of the trunk, neck, and head, and (4) rotational behavior. Animals were scored every 30 minutes for 2 hours after treatment. The frequency and intensity of AIMs were evaluated using the following rating: 0, absent; 1, occasional; 2, frequent; 3, continuous but interrupted by external stimuli; and 4, continuous and not interrupted by external stimuli. Rotational tests were carried out in a cylinder and only completed turns (360°) were counted. All behavioral tests were done in a blinded fashion. Two hours after the last L-DOPA injection, the animals were sacrificed for immunocytochemistry and western blotting analyses.

### 2.4. Tissue Preparation and Immunocytochemistry

After completing the above behavioral tests, rats (*n* = 4) were killed under anesthesia and immediately perfused transcardially with 4% paraformaldehyde (dissolved in PBS). The brains were fixed in 4% buffered formalin and embedded in paraffin for sectioning. Coronal sections (5 *μ*m) were cut and stained with hematoxylin/eosin (H&E) for histopathological evaluation. Fixed brain sections were deparaffinized and hydrated. After antigen-retrieval in boiling 10 mM sodium citrate for 20 min, sections were incubated in rabbit polyclonal anti-FosB (1 : 2000, Santa Cruz, CA, USA) overnight at 4°C and then incubated in the avidin-biotin peroxidase complex using the appropriate rabbit ABC Vectastain kit (Vector Laboratories, USA). Immunoreactivity was observed using 3, 3-diaminobenzidine (DAB, Sigma, USA) as the chromagen. Positive immunoreactive cells were counted under the microscope in five randomly chosen high-magnification fields (×200) in the striatal region and analyzed using the software Imagine J by an independent experimenter blind to the sections.

### 2.5. Western Blotting

After anesthesia with 10% chloral hydrate of the remaining rats, tissue samples harvested from the bilateral dorsal striatum were extracted with lysis buffer containing protease inhibitors, homogenized, and centrifuged. Supernatants yielding 30 *μ*g protein were subjected to 10% SDS-polyacrylamide gels and electrophoretically transferred to polyvinylidene difluoride (PVDF) membranes. Membranes were incubated with specific primary antibodies: polyclonal goat-anti phosphor-DARPP-32 at threonine34 (1 : 100, Santa Cruz), polyclonal rabbit anti-DARPP-32 (1 : 200, Santa Cruz), polyclonal rabbit anti-FosB (1 : 200, Santa Cruz), polyclonal rabbit anti-ERK1/2 phosphorylated at threonine202/tyrosine204 of ERK1, and threonine185/tyrosine187 of ERK2 (1 : 1000, Cell Signaling Technology), followed by incubation with horseradish peroxidase-conjugated secondary antibody (ICN Pharmaceuticals). Proteins were visualized with a Super Signal West Pico chemiluminescence kit (Thermo Scientific, USA). *β*-actin (1 : 500, Santa Cruz) was used to normalize protein expression.

### 2.6. Statistical Analysis

Statistical analysis was performed using SPSS 16.0 software. For AIMs, comparison between groups was made using nonparametric test. For immunocytochemistry and western blotting analysis, Student's *t*-tests were used when only two groups were compared. The differences between groups (more than two groups) were made by two-way analysis of variance (ANOVA) followed by post hoc comparison with Bonferroni test depending on the data. Significance level was set at *P* < 0.05. Data are represented as mean ± SEM.

## 3. Results

### 3.1. Behavioral Effects (Levetiracetam Reversed AIMs in a Dose-Dependent Manner)

L-DOPA treatment induced AIMs that are dyskinetic-like movements in hemiparkinsonian rats, as expected in the unilateral 6-OHDA-lesion model. The chronic administration of L-DOPA 12 mg/kg twice daily induced gradual development of AIMs over the course of the treatment period. The incidence and intensity of AIMs were maximal from 30 to 60 min after L-DOPA injection. AIMs scores decreased gradually to reach baseline after 2 to 3 hours from injection. The frequency and severity of AIMs increased significantly during the first 5 to 12 days of L-DOPA treatment. After that time, their intensity remained stable (Figure 1(a)).

By the 5th day of treatment, all levetiracetam doses significantly reduced the total AIMs and forelimb scores compared to the control group, and this effect was maintained until the end of the L-dopa response (Figures 1(a) and 1(b)). Levetiracetam reduced the severity of AIMs particularly in the forelimbs. The effects of levetiracetam 30 and 60 mg on forelimb AIMs were significant in the 3rd day of treatment with score reduction from 2.13 ± 0.13 to 1.5 ± 0.18 and 1.17 ± 0.11, respectively (*n* = 10/group, *P* < 0.05, Figure 1(b)). Levetiracetam also reduced orolingual dyskinesia and axial dystonia since the 7th day of treatment, but only at the 60 mg dose (Figures 1(c) and 1(d)). The total AIMs scores reduced from 8.08 ± 0.74 to 6.92 ± 0.37 in the 30 mg levetiracetam group (*P* < 0.05, *n* = 10/group, Figure 1(a)) and to 6.42 ± 0.58 in the 60 mg levetiracetam group (*n* = 10/group, *P* < 0.01). The lowest dose of levetiracetam (15 mg) had a smaller effect on AIMs during the first 10 days of treatment and became nonsignificant after the 11th day. These data indicate a dose-dependent effect of levetiracetam on AIMs.

Levetiracetam did not interfere with the antiparkinsonian action of L-DOPA. Contralateral rotation, a measurement of such an L-Dopa action, increased during the first week of daily L-DOPA treatment and stabilized in the second week with no differences across the control and levetiracetam treatment groups (Figure 1(e)). These behavioral data are aligned with previous reports showing no differences in Parkinsonian motor disability or “on-time” effect [14]. These results indicate that levetiracetam effect on AIMs is not related to diminishment in the beneficial antiparkinsonian action of L-DOPA.

### 3.2. Molecular Effects (Levetiracetam Decreased the Expression of ΔFosB and the Phosphorylation of DARPP-32 and ERK1/2 in the Striatum)

**Figure 2:**
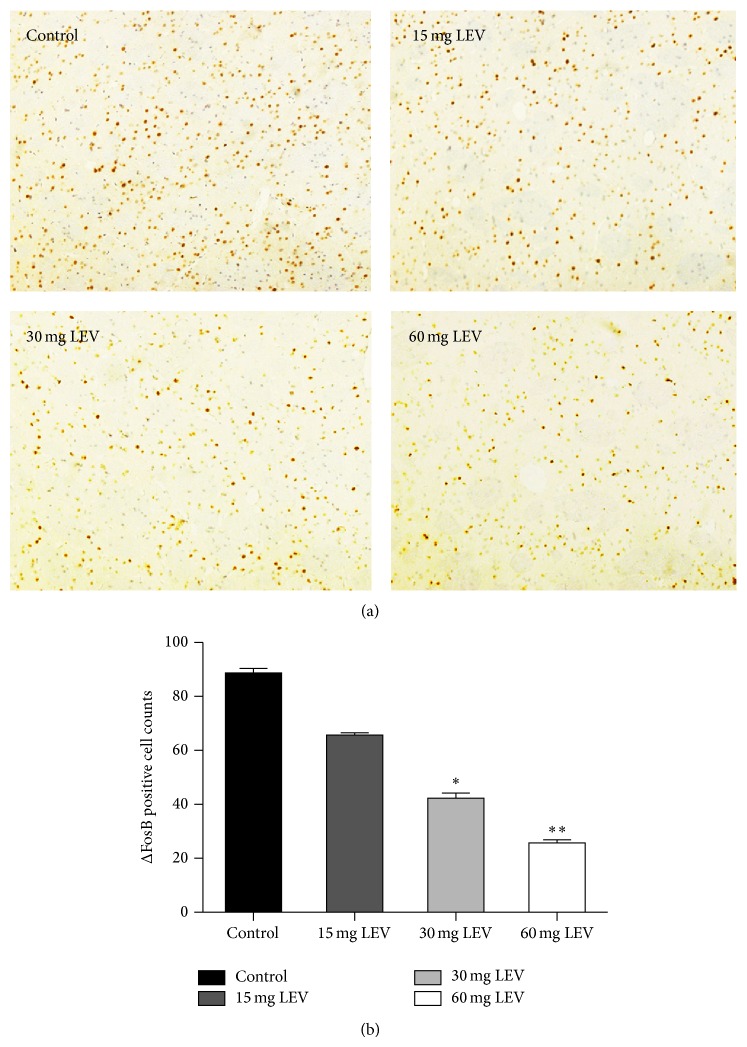
Levetiracetam-induced changes in FosB/ΔFosB immunostaining of the dorsolateral portion of the striatum. (a) Images of striatal slices from the lesion side of an animal from each treatment group (control, 15, 30, and 60 mg/kg levetiracetam denoted as LEV; ×200). (b) Immunoreactive cell count in each treatment group. Values are mean ± SEM. ^*^ *P* < 0.05 and ^**^ *P* < 0.01 versus control group (*n* = 4 per group).

FosB/ΔFosB immunoreactive neurons increased in the dorsolateral part of the striatum on the lesion side with the used antibody that recognizes all members of the FosB family. All doses of levetiracetam decreased the number of FosB/ΔFosB positive cells (from 88.7 ± 1.7/section in the control group to 65.7 ± 0.87, 42.3 ± 1.88, and 25.7 ± 1.2/section in the 15, 30, and 60 mg groups, resp.; Figure 2). These results indicate dose-dependent effects of levetiracetam on FosB/ΔFosB expression.

**Figure 3:**
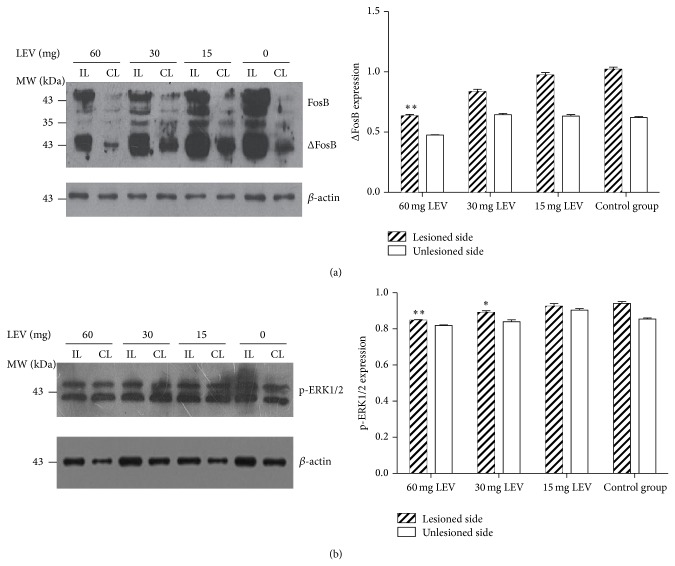
Levetiracetam-induced expression changes of the ΔFosB and phosphorylation of ERK1/2 in the dorsolateral portion of the striatum. Images display immunoblots from striatal tissue (IL: ipsilateral side; CL: contralateral side) from one animal in each levetiracetam treatment group, and immunoblots correspond to FosB, ΔFosB, and *β*-actin as internal control. Levetiracetam 60 mg/kg significantly reduced the striatal ΔFosB expression (a). ^#^ *P* < 0.05 versus same side of control group (*n* = 6 per group; LEV: levetiracetam). Images display immunoblots from striatal tissue (IL: ipsilateral side; CL: contralateral side) from one animal in each levetiracetam treatment group, and immunoblots correspond to p-ERK1/2 and *β*-actin as internal control. Levetiracetam 30 and 60 mg/kg significantly reduced the striatal p-ERK1/2 expression (b). ^*^ *P* < 0.05 and ^**^ *P* < 0.01 versus same side of the control group (*n* = 6 per group; LEV: levetiracetam).

To determine the selective effects of levetiracetam on ΔFosB levels, striatal tissue was processed for immunoblotting as well. The striatal expression of ΔFosB, the truncated form of FosB, was exaggeratedly higher in the lesioned side compared to the intact side of the striatum after chronic L-DOPA treatment (*P* < 0.01, Figure 3(a)). All doses of levetiracetam reduced striatal ΔFosB levels from 1.02 ± 0.02 in the control group to 0.97 ± 0.02 (*P* < 0.05), 0.83 ± 0.02 (*P* < 0.05), and 0.64 ± 0.01 (*P* < 0.01) in the 15, 30, and 60 mg levetiracetam treatment group, respectively.

The striatal expression of p-ERK1/2 also increased in the lesioned side compared to the intact side of the striatum after chronic L-DOPA treatment (*P* < 0.01, Figure 3(b)). Levetiracetam 30 and 60 mg reduced the striatal expression of p-ERK1/2 (from 0.94 ± 0.01 to 0.89 ± 0.01 and 0.84 ± 0.003, resp., *P* < 0.05).

**Figure 4:**
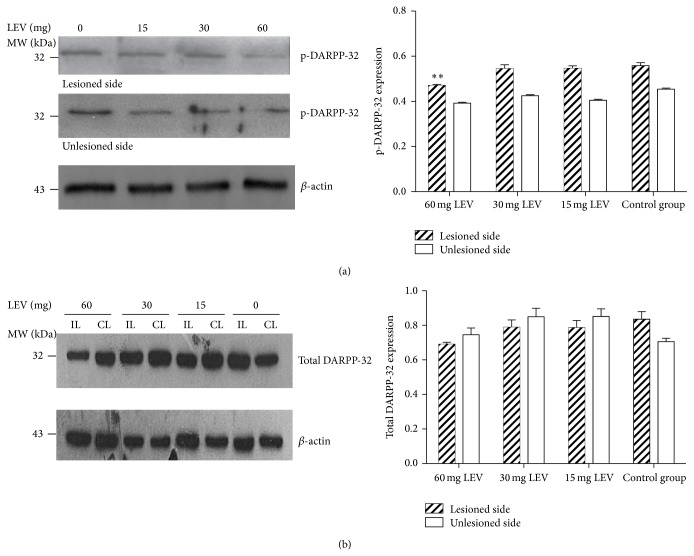
Levetiracetam-induced changes in the phosphorylation of DARPP-32 in the dorsolateral portion of the striatum. (a) Images display immunoblots from striatal tissue (lesioned and unlesioned side) from one animal in each levetiracetam treatment group. Immunoblots correspond to p-DARPP-32 and *β*-actin as internal control. The graph compares p-DARPP-32 expression across treatment groups. Levetiracetam 60 mg/kg significantly reduced the striatal p-DARPP-32 expression. ^**^ *P* < 0.01 versus same side of the control group. (b) Images display immunoblots from striatal tissue (IL: ipsilateral side; CL: contralateral side) from one animal in each levetiracetam treatment group. Immunoblots correspond to total DARPP-32 and *β*-actin as internal control. The graph compares total DARPP-32 expression across treatment groups. Levetiracetam had nonsignificant effects on the striatal total DARPP-32 expression (*n* = 6 per group; LEV: levetiracetam).

DARPP-32 phosphorylation at threonine34 increased in the lesioned side compared to the intact side of the striatum (*P* < 0.01, Figure 4(a)). Levetiracetam 60 mg significantly reduced the striatal expression of p-DARPP-32 (0.56 ± 0.01 versus 0.47 ± 0.01, *P* < 0.01). Lower doses of levetiracetam (15 and 30 mg) had little effect on the phosphorylation of DRAPP-32. Levetiracetam had no effect on total DARPP-32 levels, which were similar between the lesioned and intact side of the striatum (*P* > 0.05, Figure 4(b)). Altogether, these data indicate that levetiracetam regulates the striatal expression of molecules involved in the mechanisms of dyskinesias, that is, ΔFosB, p-DARPP-32, and p-ERK1/2.

## 4. Discussion

In the present study, we found that levetiracetam reduced the development of AIMs, the rodent equivalent of LID, in hemiparkinsonian rats following chronic exposure to L-DOPA, and these results in rodents are in agreement with the previous study in MPTP-lesioned macaques showing effects on established LID as well as L-DOPA priming for LID [14, 15]. The development of AIMs was associated with increased ΔFosB expression and p-ERK1/2 and DARPP-32 phosphorylation in the striatum ipsilateral to the lesion. The most striking change was in ΔFosB levels that increased 2-3 fold in the denervated side compared to the intact side. Our data showed that the molecular changes associated with LID were reversed by levetiracetam, and it should be noted that the effects of LVT appear limited.

The exact pathogenetic mechanisms underlying LID are still unclear; however, the evidence indicates that exogenous L-DOPA given with a pulsatile regimen is converted to dopamine irregularly in the remaining dopamine terminals and neighbor cells leading to nonphysiologic dopamine release in the striatum and thereby fluctuating stimulation on postsynaptic dopamine receptors [19–21]. This abnormal stimulation may cause imbalance of dopamine responses in striatal output neurons [3]. Studies in rodent and primate models have shown that dopamine depletion followed by the subsequent replacement therapy is associated with hypersensitive responsiveness of striatal neurons expressing the dopamine D_1_ receptors and projecting into the direct output pathway [6, 22]. D_1_ receptor activation increases cyclic adenosine 3′,5′-monophosphate (cAMP), which in turn activates cAMP-dependent protein kinase A (PKA). This enzyme is critical for the phosphorylation of various proteins downstream in the signaling cascade. One of these proteins is DARPP32, its phosphorylation at the threonine-34 residue is induced by L-DOPA through D_1_ receptor-PKA-mediated mechanisms. The striatal levels of phospho[Thr34]-DARPP-32 were found to correlate with the severity of dyskinesia in rodents [22, 23], and deletion of DARPP-32 gene significantly reduced AIMs. In addition, DARPP-32, through 2 mitogen-activated protein kinases (MAPKs), ERK1 and ERK2, participates in the regulation of gene expression in the striatum of dyskinetic animals [7, 8]. Following chronic L-DOPA administration in rats and monkeys, p-ERK1/2 is elevated in striatal medium spiny neurons directly correlating with LID severity, and LID can be attenuated by reducing striatal p-ERK1/2 levels [24]. Other important molecule participating in the mechanisms of dyskiniesias is ΔFosB, a truncated splice variant of the immediate early gene FosB [9]. Striatal ΔFosB expression increases significantly following dopamine denervation, and it elevates to even higher levels with chronic L-DOPA treatment in rats and macaques correlating with dyskinesia severity [10, 13, 25]. Furthermore, ΔFosB overexpression has been observed in postmortem striatal studies of Parkinsonian patients chronically treated with L-DOPA [26].

Our data showed that levetiracetam had specific effects on striatal ΔFosB, p-DARPP-32, and p-ERK1/2, and these molecular effects were parallel to the LID changes. Also both the molecular and behavioral effects were dose-dependent. Levetiracetam is a new type of antiepileptic drug, which binds to ion channels and specifically to the presynaptic vesicle protein SV2A participating in neurotransmitter release. It has been proved efficient as adjunctive therapy in patients with partial seizures [27]. In mechanistic studies, it was found that this antiepileptic drug can activate the ROMK1 channel through PKA phosphorylation as well [28]. An effect on PKA phosphorylation may also be involved in the antidyskinetic actions of levetiracetam. It is plausible that levetiracetam acts at multiple sites in the molecular pathways associated with LID development [29]. Of note, the most prominent effect of levetiracetam was the reduction of ΔFosB expression, which cannot be explained by any of its known actions on vesicular protein or ion channels. Therefore, the exact mechanism(s) underlying the antiepileptic effects of levetiracetam remains uncertain. The present study revealed that levetiracetam induces striatal molecular changes that are tightly linked to the occurrence of LID and, therefore, levetiracetam can specifically elicit antidyskinetic effects.

Several open-label clinical trials have confirmed efficacy of levetiracetam for the management of levodopa-induced dyskinesias in PD [30, 31]. In addition, in a randomized, double-blind, placebo-controlled, pilot study in patients with moderate-to-severe LID, levetiracetam provided significant improvement of dyskinesias [32]. As in these limited clinical trials, the present study and other tests in animal models showed that levetiracetam has antidyskinetic effects without affecting the antiparkinsonian action of L-DOPA [33]. This pharmacological profile together with its specific effects on molecular mediators of LID supports the therapeutic potential of this drug and others in the same class for PD. Furthermore, the present data support further studies into the mechanisms of action of levetiracetam, particularly to investigate its role on modulation of striatal PKA signaling, corticostriatal plasticity and the abnormal medium spiny neuron activity in association with dyskinesias [3, 6, 34].

## 5. Conclusion

In summary, we have demonstrated that Levetiracetam can attenuate AIMs dose-dependently in 6-hydroxydopamine lesioned rats. Levetiracetam reverses the striatal ΔFosB overexpression and reduces the striatal levels of phosphorylated ERK1/2 and DARPP-32, associated with the improvement of LID. This study supports that levetiracetam acts at multiple sites in the pathogenetic cascade of LID and that further understanding of these actions of antiepileptics may contribute to develop new LID therapies.
